# Self-medication in children aged 0–12 years in Brazil: a population-based study

**DOI:** 10.1590/1984-0462/2024/42/2022137

**Published:** 2023-07-10

**Authors:** Emilia da Silva Pons, Tatiane da Silva Dal Pizzol, Daniela Riva Knauth, Sotero Serrate Mengue

**Affiliations:** a Médicos sem Fronteiras Brasil, Rio de Janeiro, RJ, Brazil.; b Universidade Federal do Rio Grande do Sul, Porto Alegre, RS, Brazil.

**Keywords:** Children, Self-medication, Prevalence, Health surveys, Brazil, Crianças, Automedicação, Prevalência, Inquéritos epidemiológicos, Brasil

## Abstract

**Objetive:**

Studies have shown that the practice of self-medicating children occurs worldwide and is independent of the country’s economic level, medication policies, or access to health services. This study aimed to estimate and characterize the prevalence of self-medication in the Brazilian population of children aged up to 12 years.

**Methods:**

We analyzed the data of 7528 children aged up to 12 years whose primary caregivers responded to the National Survey on Access, Use and Promotion of Rational Use of Medicines in Brazil (PNAUM), a cross-sectional population-based study conducted in 245 Brazilian municipalities. The prevalence of self-medication was defined as the use of at least one medication without a doctor’s or dentist’s indication 15 days before the interview.

**Results:**

The prevalence of self-medication was 22.2% and was more frequent in older children belonging to poorer families and without health insurance. The acute conditions for which there was a higher frequency of self-medication were pain, fever, and cold/allergic rhinitis. Analgesics/antipyretics stood out among the most used medications for self-medication.

**Conclusions:**

The prevalence of self-medication to treat acute conditions was high in Brazilian children sampled in PNAUM, emphasizing the management of common symptoms such as pain, fever, and cold/allergic rhinitis in this age group. These findings reinforce the need for educational actions aimed at parents and caregivers.

## RESUMO

**Objetivo:**

Estudos têm mostrado que a prática de automedicar crianças ocorre mundialmente e independe do nível econômico do país, das políticas de medicamentos ou do acesso aos serviços de saúde. O objetivo deste estudo foi estimar e caracterizar a prevalência de uso de medicamentos por automedicação na população brasileira de crianças de zero a 12 anos de idade.

**Métodos:**

Foram analisadas informações de 7.528 crianças de zero a 12 anos cujo cuidador principal respondeu à Pesquisa Nacional sobre Acesso, Utilização e Promoção do Uso Racional de Medicamentos no Brasil (PNAUM), estudo transversal de base populacional realizado em 245 municípios brasileiros. A prevalência de automedicação foi definida como o uso de pelo menos um medicamento sem indicação de médico ou dentista nos 15 dias anteriores à entrevista.

**Resultados:**

A prevalência de automedicação foi de 22,2% e foi mais frequente nas crianças mais velhas e pertencentes a famílias mais pobres e sem plano de saúde. As condições agudas para as quais houve maior frequência de automedicação foram dor, febre, resfriado e rinite alérgica. Analgésicos/antipiréticos destacaram-se entre os medicamentos mais utilizados por automedicação.

**Conclusões:**

A prevalência de automedicação no manejo de condições agudas foi elevada nas crianças brasileiras amostradas na PNAUM, com destaque para o manejo de sintomas comuns nessa faixa etária, como dor, febre, resfriado e rinite alérgica. Esses achados reforçam a necessidade de ações educativas voltadas aos pais e cuidadores.

## INTRODUCTION

The use of drugs in children requires special attention due to uncertainties regarding the efficacy and safety of the products. These uncertainties stem from the scarcity of clinical trials in this population for ethical, legal, and economic reasons. Hence, the use of drugs in children is mainly based on adaptations of the regimens for adults, on information obtained from observational studies and expert consensus.^1^

Despite the risks and uncertainties of the use of medicines in the child population, self-medication in children, understood as the administration, by the caregiver, of medicines without an indication from a health professional, has been described as a common phenomenon of interest to the health public.^2,3,4,5^ Self-medication is not an autonomous, free, and voluntary decision in pediatry, with rare exceptions. Self-medication in children is not based on their perceptions and interpretations of symptoms, but on the subjective understanding of the mother or other caregiver,^6^ which makes the phenomenon complex.

Although self-medicating children is frequent, updated epidemiological data are scarce.^2^ The few studies available have shown that the practice is worldwide and is independent of the country’s economic level, medication policies, or access to health services.^3,7,8^

Differences in study designs, sample selection, age groups, and recall period result in very different frequencies of self-medication. In a review by Pfaffenbach et al.,^3^ which included studies carried out in Brazil, China, Portugal, Spain, and Nigeria, the prevalence of self-medication in children and adolescents was greater than 20%, ranging from 21.1 to 67.7%. In a study carried out in Germany, the prevalence of self-medication in the population aged between 0 and 17 years was 25.2%, corresponding to 38.5% of all medications used.^2^ In Finland, a study carried out with a representative sample of the population of children aged under 12 years resulted in a prevalence of self-medication of 50%.^9^

In Brazil, Pereira et al.^5^ described a prevalence of self-medication of 56.6% in children and adolescents aged up to 18 years living in two cities in the interior of the state of São Paulo. Silva and Giugliani found that more than half of the statements of drug consumption by school adolescents in Porto Alegre (RS), in the 7 days before the survey (53.2%), had no medical indication.^10^ In another study, carried out with a cohort of children born in 2004 and living in a city in the interior of Rio Grande do Sul, self-medication increased as a function of age, with 11, 26, and 34% at 3, 12, and 24 months, respectively.^11^ In a more recent study, Farías-Antunéz et al. noted a decrease in the proportion of medically prescribed medications between ages 3 and 48 months and an increase in self-medication.^12^ We did not find population-based studies with national coverage that evaluated self-medication exclusively in children.

This study aimed to estimate and characterize the prevalence of over-the-counter medication use in Brazilian children aged 0–12 years. As a secondary objective, we evaluated the active substances most used by self-medication in this group.

## METHOD

This study is based on the data collected by the National Survey on Access, Use and Promotion of Rational Use of Medicines in Brazil (PNAUM), conducted between September 2013 and January 2014. This survey is a population-based cross-sectional study with a probabilistic sample in three stages. The first stage corresponds to the municipalities, the second stage is census tracts, and the third stage represents the households. The study population consisted of residents in permanent private households in the urban area of Brazil. A total of 245 municipalities were included, and 41,433 people were interviewed, corresponding to the five geographical regions of Brazil. The sample size was defined based on the estimates of access and use of medicines obtained from previous studies.

A total of 165 interviewers conducted the face-to-face interviews, and data were collected using tablets with 3G and GPS connections. The data analyzed in this study are limited to the information of 7,528 children aged 0–12 years whose primary caregivers responded to the PNAUM, which comprises 18.2% of the sample. Additional details of the PNAUM are described in a methodological article.^13^

The data collection instruments were developed by researchers from seven Brazilian universities, having been tested and standardized before implementation. The questions were adapted to be answered by the child’s primary caregiver. The complete questionnaires can be accessed in the Ministry of Health publication.^14^

The prevalence of medication use in the last 15 days to treat acute conditions was calculated from the answers to the questions: “In the last 15 days, did the <<child’s name>> use any medication for infection; nausea and/or vomiting; stomach or bowel problems; fever; diarrhea; pain; flu, cold or allergic rhinitis?” The use of vitamins was also investigated through the question, “In the last 15 days, did <<child’s name>> use any vitamin, mineral supplement, appetite stimulant, or tonic?” For this analysis, such products were considered medicines. Finally, any other medication in the last 15 days that had not yet been mentioned during the interview was investigated. To avoid possible biases, caregivers were asked to present, whenever possible, the packaging or prescription of the medication used.

The use of medicines without a doctor’s or dentist’s indication, defined in this study as self-medication, following the National Medicines Policy, was investigated through the question, “Who recommended this medicine to << child’s name>>?” (doctor or dentist; pharmacist; other health professionals; and self-employed). If the caregiver answered “pharmacist,” “other health professionals,” or “self-employed,” we classified the use as self-medication. We did not ask the caregiver if the doctor’s or dentist’s indication was written or only verbal, current, or previous. We defined the prevalence of self-medication as the use of at least one medication without a doctor’s or dentist’s indication 15 days before the interview. The denominator corresponds to the number of children who used it in the last 15 days.

Children were characterized by sociodemographic, economic, and health-related factors (independent variables). These variables included: sex (male or female); age (<2 years, 2–5 years, or 6–12 years); economic class (AB, C, or D/E), according to the Brazilian Economic Classification Criteria, of the Brazilian Association of Research Companies (ABEP);^15^ health insurance (yes or no); primary caregiver (mother, grandmother/grandfather, father, or others); and caregiver’s education (0–8 years, 9–11 years, or 12 years or more).

The variables were represented by relative frequencies and their respective 95% confidence intervals (95%CI) in all analyses. Associations between independent variables and self-medication were estimated using the prevalence ratio (PR), with 95%CI. A Poisson regression model was used to estimate crude and adjusted PRs. In the first stage, the independent variables were analyzed individually. In the univariate analyses, those that presented statistical significance, defined as p<0.20, were selected for the second stage, in which all variables were introduced in the multiple model. The variables that presented p>0.05 at this stage were removed one by one from the model with a “backward” selection of the variables, leaving only the variables with p<0.05 in the final model. SPSS statistical software version 18.0 for Windows was used.^16^

The National Research Ethics Committee approved the project (CONEP — Opinion 398,131, September 16, 2013). The child’s primary guardian signed a two-way consent form before responding to the interview.

## RESULTS

The characteristics of the children participating in the PNAUM are shown in Table 1. There was a predominance of children aged between 6 and 12 years, belonging to economic class C and without health insurance coverage. The primary caregivers that answered the questionnaire were children’s mothers (76.1%).

**Table 1.** Characteristics of the children participating in the PNAUM. PNAUM, Brazil, 2014 (n=7,528).

Characteristics	Prevalence %	95%CI
Sex		
Male	49.7	47.6–51.7
Female	50.3	48.3–52.4
Age (years)		
<2	13.5	12.7–14.4
2–5	26.9	25.4–28.4
6–12	59.6	57.8–61.4
Economic class (ABEP)		
A/B	16.8	15.3–18.4
C	56.2	54.2–58.2
D/E	27.0	25.3–28.8
Health insurance		
Yes	19.3	17.7–20.9
No	80.7	79.1–82.3
Primary caregiver		
Mother	76.1	74.4–77.8
Grandmother/grandfather	9.6	8.5–10.8
Father	8.6	7.6–9.7
Other	5.7	4.7–6.8
Primary caregiver’s education		
0–8 years	48.8	46.8–50.8
9–11 years	41.6	39.5–43.6
12 years or more	9.6	8.6–10.8

PNAUM: National Survey on Access, Use and Promotion of Rational Use of Medicines in Brazil; ABEP: Brazilian Association of Research Companies.

Table 2 shows the prevalence of medication use and self-medication in the 15 days before the interview. The age group under 2 years had a higher prevalence of medication use but a lower prevalence of self-medication than older children. There is also a higher prevalence of self-medication in children without health insurance coverage and among those whose mother or grandmother/grandfather are the primary caregivers. In the crude and adjusted analyses (Table 3), older age, lower socioeconomic class, and lack of health insurance showed a positive and statistically significant association with self-medication. A negative and statistically significant association between caregivers’ higher education and self-medication, verified in the crude analysis, was not maintained in the adjusted analysis. The other variables (gender and primary caregiver) were not associated with the outcome analyzed.

**Table 2.** Prevalence of medication use and self-medication in the 15 days before the interview by children participating in the PNAUM. PNAUM, Brazil, 2014 (n=7,528).

Characteristics	Prevalence of medication use* (%)	95%CI	Prevalence of self-medication^†^ (%)	95%CI
Sex				
Male	28.0	25.0–31.3	21.6	18.4–25.2
Female	26.1	23.5–28.9	22.7	19.1–26.8
Age (years)				
<2	46.5	43.3–49.7	16.4	14.1–19.0
2–5	32.9	30.0–35.8	20.3	17.7–23.3
6–12	20.0	17.4–22.9	26.6	21.6–32.2
Economic class (ABEP)				
A/B	22.7	18.9–27.0	17.1	11.6–24.5
C	27.6	24.8–30.6	23.3	20.1–26.9
D/E	28.5	24.8–32.5	22.6	18.2–27.6
Health insurance				
Yes	32.2	27.9–36.9	11.9	8.0–17.3
No	25.8	23.41–28.3	25.2	22.4–28.3
Primary caregiver				
Mother	29.4	26.9–32.0	22.3	19.7–25.2
Grandmother/grandfather	20.0	15.8–25.1	25.8	17.2–36.7
Father	19.0	14.5–24.4	18.4	11.6–27.9
Other	19.6	12.2–29.8	18.5	7.7–38.3
Primary caregiver’s education				
0–8 years	22.9	20.2–25.9	22.2	18.5–26.4
9–11 years	31.1	27.1–35.3	23.8	20.0–28.1
12 years or more	32.6	27.0–38.8	20.1	13.2–29.5
Total	27.1	24.8–29.4	22.2	18.9–25.8

PNAUM: National Survey on Access, Use and Promotion of Rational Use of Medicines in Brazil; ABEP: Brazilian Association of Research Companies.*n=2,732 children used at least one drug in the last 15 days to treat acute conditions; ^†^n=689 children used at least one self-medication drug in the last 15 days to treat acute conditions.

**Table 3.** Crude and adjusted prevalence ratios of self-medication, according to demographic and socioeconomic aspects. PNAUM, Brazil, 2014 (n=7,528).

Characteristics	Crude PR	95%CI	Adjusted PR	95%CI
Sex				
Male	1.00	0.88–1.14		
Female	1			
Age (years)				
<2	1		1	
2–5	1.29	1.12–1.49	1.29	1.12–1.48
6–12	1.54	1.27–1.88	1.57	1.29–1.91
Economic class (ABEP)				
A/B	1			
C	1.39	1.09–1.77	1.18	0.93–1.50
D/E	1.70	1.32–2.18	1.36	1.06–1.75
Health insurance				
Yes	1		1	
No	2.29	1.80–2.91	2.13	1.66–2.73
Primary caregiver				
Mother	1			
Grandmother/grandfather	0.97	0.64–1.45		
Father	0.77	0.58–1.03		
Other	0.93	0.73–1.19		
Primary caregiver’s education				
0–8 years	1			
9–11 years	0.85	0.70–1.03		
12 years or more	0.68	0.53–0.86		

PNAUM: National Survey on Access, Use and Promotion of Rational Use of Medicines in Brazil; ABEP: Brazilian Association of Research Companies.

The prevalences of medication use and self-medication due to the disease or condition treated are shown in Table 4. There is a predominance of medication use for fever management (30.9%). Self-medication for pain relief (28.7%) was the most frequent among the health conditions investigated.

**Table 4.** Prevalence of medication use and self-medication in the 15 days prior to the interview, stratified by acute illness or treated condition in children participating in the PNAUM. PNAUM, Brazil, 2014.

Treated disease or condition and use of vitamins, supplements, and tonics	Medication use		Self-medication	
	%	95%CI	%	95%CI
Infection	16.4	14.2–19.0	14.4	10.3–19.9
Cold or allergic rhinitis	26.9	24.2–29.7	24.6	20.2–29.6
Gastrointestinal problems*	9.2	7.6–11.1	22.1	15.5–30.5
Fever	30.9	28.0–34.1	24.6	20.4–29.3
Pain	20.9	18.2–24.0	28.7	22.2–36.2
Vitamin, mineral supplement, appetite stimulant, or tonic	22.6	20.3–25.0	21.6	17.6–26.3

PNAUM: National Survey on Access, Use and Promotion of Rational Use of Medicines in Brazil. *Including nausea, vomiting, and diarrhea.

The 10 most used active substances for self-medication are dipyrone (19.7%), paracetamol (12.7%), ibuprofen (5.2%), ascorbic acid (3.4%), ambroxol (2.6%), trimethoprim/sulfamethoxazole (2.2%), dexchlorpheniramine (2.2%), nimesulide (2.2%), amoxicillin (2.1%), and acetylsalicylic acid (1.9%). Approximately 30% of all drugs used without prescription corresponded to analgesics/antipyretics.

## DISCUSSION

More than one-fifth of the children who used drugs for acute conditions did so without a doctor’s or dentist’s indication (22.2%). Studies carried out in the country explored this theme with samples limited to one,^11,17^ two municipalities,^5^ or a region,^1^ with a prevalence of self-medication ranging from 11 to 56.6%.

However, differences in study designs and age groups make comparisons with each other and with this study difficult. Few international studies have addressed self-medication in samples of the population of children. In a national survey carried out in Germany with 17,450 children and adolescents aged between 0 and 17 years, 25.2% of the participants used medication for self-medication in the week before the interview.^2^ In another national mail survey of caregivers of 4,032 Finnish children, the prevalence of child self-medication in the previous 2 days was 50%.^9^

The increase in the prevalence of self-medication as the age of children increases was observed in this study and previous studies,^6,11,12^ although it is not constant. In a study carried out in Finland, self-medication was more common in children aged under 3 years and decreased with age.^9^ In turn, a study in Brazil showed that self-medication in children follows a U-shaped curve concerning age, with the highest prevalence in early life (up to 3 months old) and mid-adolescence (15 years) compared with 6 months, 12 months, 4 years, and 11 years old.^17^ In our study, the lower frequency of self-medication among children aged under 2 years could be explained by the caregiver’s perception of the greater vulnerability of younger children. Hence, medication use in this age group predominantly occurs under prescription, as already discussed by Ortiz et al.^6^ On the contrary, self-medication in adolescents is a concern due to the possibility of predicting behaviors including the inadequate pattern of drug use or abuse and determining its continuity in adulthood.^18^

In this study, children covered by health insurance had a lower prevalence of self-medication when compared to those who did not have a plan. This factor can also contribute to the higher prevalence of medication used to treat acute medical conditions. The greater contact with health services provided by the health insurance would allow new prescriptions, which may explain the higher prevalence of medication use by these children (corresponding for the most part to prescribed drugs) and the lower prevalence of self-medication. These data can also be understood from the difficulty of access to health professionals among those responsible for children without health insurance. This difficulty of access, including geographical barriers to basic health units and emergency care units, can lead to self-medicating children to solve a health problem.^19^

The mother’s level of education and the family’s socioeconomic level have also been associated with children’s self-medication. The univariate analysis found a negative association between self-medication and schooling (12 years of education or more), which was not maintained in the adjusted analysis. On the contrary, we found that children from families with lower socioeconomic status (economic class C, D, or E) had a higher prevalence of self-medication. This association was maintained in the adjusted analysis for classes D and E. The observed availability of health insurance can partly explain these results. Mothers belonging to the more privileged socioeconomic classes have greater use of health insurance to meet the health demands of their children.

Consequently, the use of medication by these children occurs primarily by prescription. This finding does not agree with the results of other studies, which indicate that the higher the mother’s level of education and the higher the family’s socioeconomic level, the greater the probability of the child receiving over-the-counter medications.^2,20,21,22^ We suggest, therefore, that self-medication in children should be considered not only based on the socioeconomic characteristics of the family but also in terms of more access to health services and professionals.

The health conditions with a higher prevalence of self-medication in children were pain, fever, and cold or allergic rhinitis. Self-medication in children is not an autonomous decision but depends on the mother’s interpretation or another caregiver. Thus, diseases that present clearly identifiable signs and symptoms, such as fever and respiratory symptoms, may favor self-medication in children. Training the mother — or another caregiver — in identifying symptoms allows her to acquire specific skills to administer a medication, which is usually considered appropriate by the doctor’s prescription in previous processes that she interprets as similar.^6^ It should be noted that some health conditions, such as fever and the common cold, are generally self-limiting, at least in healthy children, for whom the care provided by the mother or other caregiver may be sufficient, provided they have the necessary skills. Among the skills, we highlight the correct identification of symptoms, monitoring their evolution, and identification of warning signs or symptoms. In addition, they should have sufficient health literacy for the proper administration of medicines, including possible dose adjustments based on the child’s age and weight and other fundamental care for the safe use of medicines. Noteworthy, the occasional use of a medicine for an acute, self-limiting condition previously evaluated by a doctor should be considered differently than self-medicating a child for a chronic health condition that was not yet been properly diagnosed by a doctor.

The active substances most used by children reflect the use of drugs to treat colds, fever, pain, and infections, which converges with the findings of studies carried out in countries such as Germany,^2^ Finland,^9^ Spain,^6^ and Romania.^23^ It is interesting to note that, although the acquisition of antimicrobials in Brazil is subject to special control and prescription retention,^24^ this class of drugs appears, in this study, among the most used active substances for self-medication in Brazilian children. The use of these antimicrobials by self-medication occurs, probably, through the use of leftovers from previous prescriptions or from other family members. Other authors have already described the high prevalence of self-medication using leftovers of earlier treatments by children.^5,6,25^

This study has some limitations. The time of year in which the data for this study were collected (spring and summer) may have underestimated the prevalence of medications typically used in the coldest months of the year, especially in the South and Southeast regions of the country. Our study was carried out between September 2013 and January 2014 and did not cover all seasons of the five regions of Brazil. In addition, the possibility of recall bias, inherent to self-reported medication use, should not be ruled out. However, the prescription or medication packaging presentation has been used as a methodological strategy to minimize this bias. Besides, we choose a short recall period (15 days), according to a previous methodological study about this issue.^26^ Finally, it would be interesting to analyze the conditions of self-medicating children concerning sociodemographic variables (age, race, and education), socioeconomic status, and health insurance of the primary caregiver. However, these variables were not collected, or there are many missings.

PNAUM was the first Brazilian population-based survey designed to assess Brazilians’ use of and access to drugs, based on a national sample. Despite the large size of our sample (7,528 children aged 0–12 years residing in municipalities of different sizes from the five regions of Brazil, including all capitals), we cannot ensure that the sample is representative of the entire Brazilian pediatric population. However, we did not find previous population-based studies with national coverage that evaluated self-medication exclusively in children.

Finally, it is noteworthy that over-the-counter medications in Brazilian children aged up to 12 years are a widespread phenomenon. This use is directly proportional to the increase in the age of children, with the lowest prevalence observed in the age group under 2 years. It is used to relieve signs and symptoms identifiable by the mother or other caregiver and cause more significant concern about the risks and discomfort of children. Moreover, as our data indicate, easier access and greater contact with health services and professionals through health insurance possibly promote less use of over-the-counter medications. There is a correspondence between prescribed and non-prescription drug use, except for gastrointestinal problems, suggesting that caregivers, when using drugs in children, have a certain familiarity with the relationship between drugs and the issues identified.

In conclusion, self-medication was prevalent, especially in older children and poorer families without health insurance. Analgesics/antipyretics stood out among the most used medications for self-medication. The findings of this study reinforce the need for educational actions aimed at parents and caregivers, focusing on self-care for self-limited health conditions, monitoring, and identifying warning signs and symptoms that require medical follow-up and appropriate use of medication. Health education can be crucial for families and communities with few financial resources and limited access to health services.

## Data Availability

The database that originated the article is available with the corresponding author.
